# Interaction of Fabry Disease and Diabetes Mellitus: Suboptimal Recruitment of Kidney Protective Factors

**DOI:** 10.3390/ijms242115853

**Published:** 2023-11-01

**Authors:** Maria D. Sanchez-Niño, Maria I. Ceballos, Sol Carriazo, Aranzazu Pintor-Chocano, Ana B. Sanz, Moin A. Saleem, Alberto Ortiz

**Affiliations:** 1 Department of Nephrology and Hypertension, IIS-Fundacion Jimenez Diaz UAM, 28040 Madrid, Spain; maria.ceballos@quironsalud.es (M.I.C.); somacaju@hotmail.com (S.C.); aranzazu.pintor@fjd.es (A.P.-C.); asanz@fjd.es (A.B.S.); 2 RICORS2040, 28040 Madrid, Spain; 3 Department of Pharmacology, School of Medicine, Universidad Autonoma de Madrid, 28029 Madrid, Spain; 4 Translational Health Sciences, Bristol Medical School, University of Bristol, Bristol BS8 1UD, UK; m.saleem@bristol.ac.uk; 5 Department of Medicine, School of Medicine, Universidad Autonoma de Madrid, 28029 Madrid, Spain

**Keywords:** fabry disease, diabetes mellitus, kidney, chronic kidney disease, inflammation, fibrosis

## Abstract

Fabry disease is a lysosomal disease characterized by globotriaosylceramide (Gb3) accumulation. It may coexist with diabetes mellitus and both cause potentially lethal kidney end-organ damage. However, there is little information on their interaction with kidney disease. We have addressed the interaction between Fabry disease and diabetes in data mining of human kidney transcriptomics databases and in Fabry (*Gla*-/-) and wild type mice with or without streptozotocin-induced diabetes. Data mining was consistent with differential expression of genes encoding enzymes from the Gb3 metabolic pathway in human diabetic kidney disease, including upregulation of *UGCG*, the gene encoding the upstream and rate-limiting enzyme glucosyl ceramide synthase. Diabetic Fabry mice displayed the most severe kidney infiltration by F4/80+ macrophages, and a lower kidney expression of kidney protective genes (*Pgc1α* and *Tfeb*) than diabetic wild type mice, without a further increase in kidney fibrosis. Moreover, only diabetic Fabry mice developed kidney insufficiency and these mice with kidney insufficiency had a high expression of *Ugcg*. In conclusion, we found evidence of interaction between diabetes and Fabry disease that may increase the severity of the kidney phenotype through modulation of the Gb3 synthesis pathway and downregulation of kidney protective genes.

## 1. Introduction

Fabry disease is an X-linked inherited metabolic disorder caused by pathogenic *GLA* gene variants leading to deficient activity of the lysosomal enzyme alpha-galactosidase A, glycolipid accumulation in multiple cell types and in the circulation, and potentially lethal kidney, heart, or central nervous system end-organ damage [1]. Phenotypic variability in the severity of target organ injury is observed even within families [2,3]. Random X chromosome inactivation results in variable severity in females. However, the phenotypic variability between males having the same *GLA* variant is poorly understood. Fabry patients are not expected to be protected from the occurrence of common non-transmissible diseases that target the same organs. Diabetes mellitus (DM) is present in up to 13% of Fabry patients, especially among older ones, a prevalence similar to the general population. Older Fabry patients usually have *GLA* variants associated with later onset disease, such as N215S. A recent report from the UK biobank identified 18 participants with *GLA* N215S and three had hypertension and DM [4]. In this regard, while severe Fabry nephropathy is uncommon in patients with N215S, the frequency of kidney failure is still around 100-fold higher than in the general population [5,6]. Furthermore, advances in therapy for Fabry disease are expected to increase life expectancy, increasing the risk of age-associated conditions such as DM [7].

Fabry disease and diabetic kidney disease (DKD) can be conceptually defined as proteinuric progressive nephropathies triggered by the accumulation of metabolites: glucose and glycosylated molecules for DM and globotriaosylceramide (Gb3/GL-3) and globotriaosylsphingosine (lyso-Gb3) for Fabry disease [1,8,9,10,11]. We hypothesized that comorbidities such as DM may modulate the phenotypic expression of Fabry disease and vice versa, Fabry disease may modulate the phenotypic expression of diabetic target organ injury. Specifically, the association of DM and Fabry disease may modulate the severity of kidney disease as compared with either disease alone.

We have now studied the impact of DM on the kidney gene expression of enzymes in the Gb3 metabolic pathway in human and murine kidney transcriptomics and the impact of DM on the kidney phenotype of Fabry mice.

## 2. Results

### 2.1. Data Mining: Influence of DM on Gb3 Pathway Gene Expression in Human DKD

We explored a potential clinical interaction between DM and Fabry disease through data mining, examining the gene expression of Gb3 metabolic pathway enzymes in human DKD in the Nephroseq database of human kidney transcriptomics [12,13,14,15]. Specifically, we explored the impact of DM on the expression of genes encoding enzymes upstream of Gb3 synthesis, such as glucosylceramide synthase (encoded by *UGCG*), which is the upstream and rate-limiting enzyme, and Gb3 synthase (encoded by *A4GALT*) (Figure S1). Additionally, we also explored the impact on acid ceramidase (encoded by *ASAH1*) that may catalyze the conversion of Gb3 into lyso-Gb3 [16,17,18], and *GLA*, which encodes alpha-galactosidase A, the enzyme which is defective in Fabry disease and whose lysosomal activity removes the galactose residues that had been added by Gb3 synthase.

The most consistent finding across kidney transcriptomics datasets of human DKD was an increased tubulointerstitial expression of *UGCG* mRNA encoding the upstream and rate-limiting enzyme glucosylceramide synthase (Table S1a). These changes may be expected to increase Gb3 precursors and in association with increased tubulointerstitial *ASAH1*, to facilitate the generation of lyso-Gb3, a soluble metabolite that causes kidney cell injury [11,19]. Indeed, glucosylceramide synthase inhibitors decreased Gb3 and lyso-Gb3 in human Fabry disease [20]. The glomerular pattern differed. Glomerular Gb3 synthase gene expression was decreased while glomerular *GLA* gene expression was increased. Both changes may limit the local generation and accumulation of Gb3 in glomeruli. Additionally, increased *UGCG* and *GLA* mRNA were correlated with lower eGFR in human DKD, i.e., the genes appear to be upregulated when kidneys are more severely injured (Table S1b). The expression of *ASAH1* decreased as the severity of injury increased. The Kidney Interactive Transcriptomics (KIT) database localized the cells potentially responsible for the changes in gene expression observed in bulk transcriptomics (Table S2). Single-cell transcriptomics data were overall coherent with bulk tissue transcriptomics. They showed a consistent increase in *UGCG* expression in multiple kidney cell types, ranging from tubular cells to glomerular cells to endothelium and leukocytes. They also identified proximal tubular cells and parietal epithelial cells as sources of increased *ASAH1* expression, and decreased expression of *A4GALT* in parietal epithelial cells and collecting duct intercalated A cells.

Overall, these human DKD data suggest that DKD may modify local Gb3 metabolic pathways in the kidney, and further support a potential interaction between DM and Fabry disease that may modify the kidney phenotype when both conditions coexist. The upregulation of *UGCG* may increase Gb3 availability, while the coordinated upregulation of *GLA* may prevent Gb3 accumulation in persons with DKD not having Fabry disease, but in Fabry disease, a lack of GLA activity may result in further Gb3 accumulation in patients with coexistent diabetes and, in the case of missense genetic variants, in the accumulation of abnormal GLA proteins that may cause endoplasmic reticulum stress.

### 2.2. Interaction of DM and Fabry Disease in Mice

Overall, human kidney transcriptomics data supported the hypothesis that coexistent DM and Fabry disease may interact to modify the kidney phenotype. Next, we evaluated this hypothesis in vivo by inducing DM by administering streptozotocin (STZ) to Fabry and wild type (WT) mice and comparing them to controls administered vehicle.

Following induction of DM, no differences were apparent between diabetic WT and diabetic Fabry mice in mean glycemia values over follow-up (WT-DM 434 ± 36 vs. Fabry-DM 417 ± 36 mg/dL, *p*-value = 0.75), insulin requirements (1.76 ± 0.33 vs. 1.60 ± 0.27 IU/day, *p*-value = 0.70) or mortality. All WT mice surviving longer than 7 days required insulin and this was the case for 12/15 (80%) diabetic Fabry mice. There was one early (before day 7 after the last streptozotocin injection) death in each group and one late (beyond day 24 after the last streptozotocin injection) death in the diabetic WT group. Two (12.5%) diabetic Fabry were sacrificed because they looked sick and were found to have kidney insufficiency for a total of 2/12 (17%) and 3/16 (19%) dead or sacrificed mice in the diabetic WT and diabetic Fabry groups, respectively.

### 2.3. Impact of DM on the Kidney Expression of Genes in the Gb3 Metabolic Pathway in Mice

**Figure 1:**
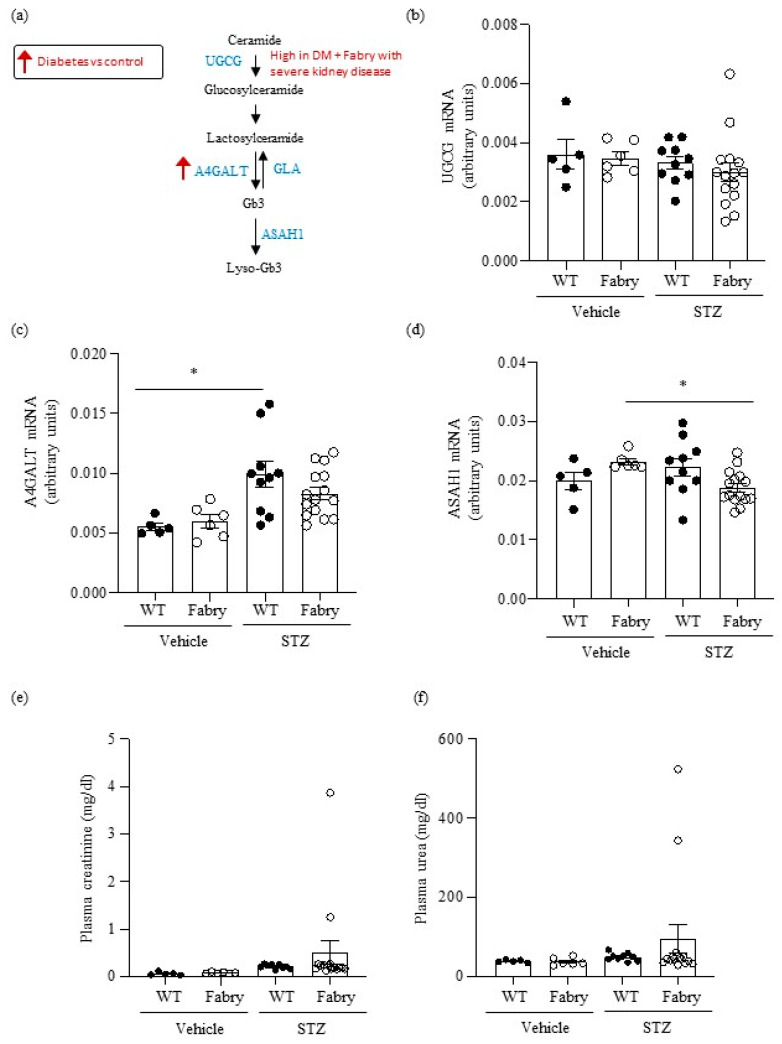
Impact of diabetes on genes encoding enzymes in the Gb3 pathway and on kidney function in wild type and Fabry mice. (**a**) Scheme of Gb3 synthesis and metabolism: glucosylceramide synthase encoded by *Ugcg*, Gb3 synthase encoded by *A4galt*, acid ceramidase encoded by *Asah1*, and alpha-galactosidase A encoded by *Gla*. Arrows present a summary of data shown in panels (**b**–**d**). (**b**–**d**) Kidney mRNA was measured by real time RT-PCR in diabetic wild-type or Fabry mice or vehicle controls. (**b**) *Ugcg*, (**c**) *A4galt*, (**d**) *Asah1*. (**e**) Plasma creatinine and (**f**) plasma urea. Data expressed as mean ± SEM of 5–15 animals per group. * *p* < 0.05 vs. respective vehicle group. Significance (*p* < 0.05) was assessed by Student’s *t-*test for two groups of data and ANOVA for three or more groups with Bonferroni post-hoc correction.

First, we explored the regulation of metabolic pathways involved in the generation of metabolites that accumulate in Fabry disease (Figure 1a). Although short-term DM in mice did not influence overall kidney *Ugcg* gene expression, very high levels were observed in two Fabry mice with DM that had developed severe kidney disease (Figure 1b), as detailed below (Section 2.7). DM also increased *A4galt* gene expression (Figure 1c), potentially further contributing to increased Gb3 availability. Diabetic Fabry mice had lower kidney expression of *Asah1* than non-diabetic Fabry mice (Figure 1d).

### 2.4. Interaction of DM and Fabry Disease on Kidney Function in Mice

Next, we addressed the potential impact of coexistent Fabry disease and DM on kidney injury. For that, we analyzed kidney function, changes in kidney gene expression and histology in diabetic WT and diabetic Fabry mice.

No differences in mean values of plasma urea and creatinine were observed between the groups (Figure 1f,e). However, 2/15 (13%) surviving diabetic Fabry mice developed kidney insufficiency characterized by high serum urea and creatinine levels, while no diabetic WT mice developed kidney insufficiency. There were no differences in median (IQR) proteinuria values at the last follow-up: 100 (30–200) mg/dL and 100 (30–200) mg/dL for diabetic WT and diabetic Fabry mice, respectively.

**Figure 2:**
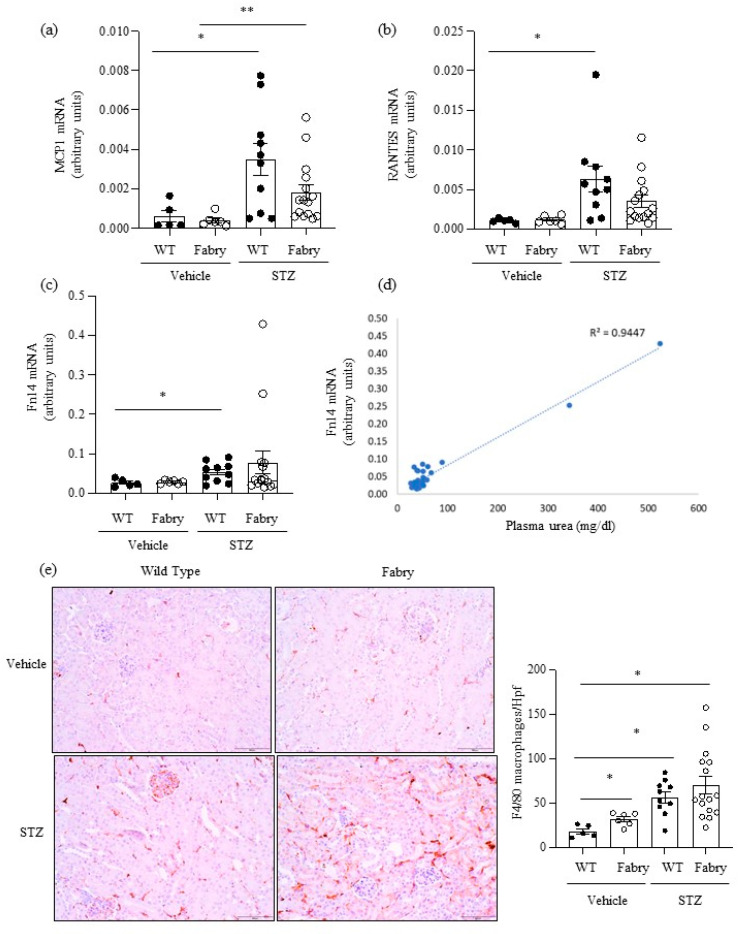
Impact of diabetes on kidney inflammation in Fabry mice. (**a**) *Mcp1*, (**b**) *Rantes* and (**c**) *Fn14* mRNA expression in kidneys was measured by real time RT-PCR. (**d**) Correlation between serum urea and kidney *Fn14* mRNA expression as assessed by linear regression. (**e**) Immunohistochemistry assessment of F4/80+ macrophages: representative images and quantitation. * *p* < 0.05 vs. vehicle WT. ** *p* < 0.05 vs. vehicle Fabry.Original magnification ×20. Data expressed as mean ± SEM of 5–15 animals per group. Significance (*p* < 0.05) was assessed by Student’s *t-*test for two groups of data and ANOVA for three or more groups with Bonferroni post-hoc correction.

**Figure 3:**
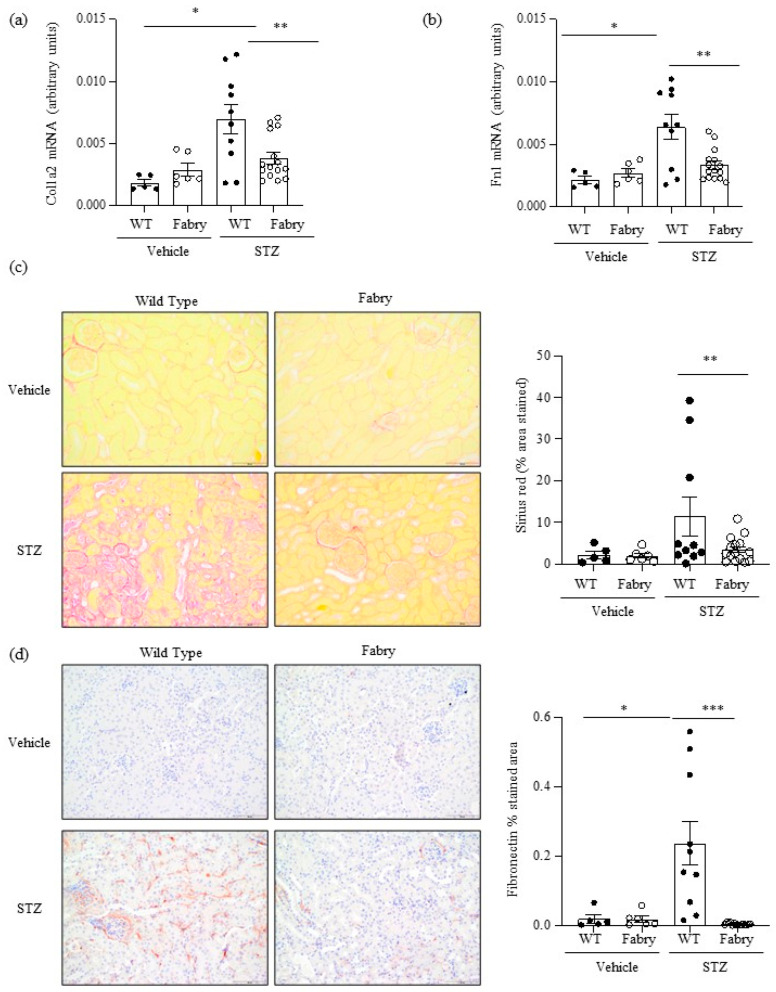
Impact of diabetes on kidney fibrosis in Fabry mice. (**a**) Kidney *Col1a2* and (**b**) *Fn1* mRNA expression was measured by real time RT-PCR. (**c**) Sirius red staining for collagen deposition: representative images and quantitation. (**d**) Immunohistochemistry disclosed milder fibronectin deposition in diabetic Fabry mice than in diabetic WT mice. Original magnification ×20. * *p* < 0.05 vs. vehicle WT. ** *p* < 0.01 vs. WT diabetic, *** *p* < 0.0005 vs. WT diabetic. Data expressed as mean ± SEM of 5–15 animals per group. Significance (*p* < 0.05) was assessed by Student’s *t-*test for two groups of data and ANOVA for three or more groups with Bonferroni post-hoc correction.

**Figure 4:**
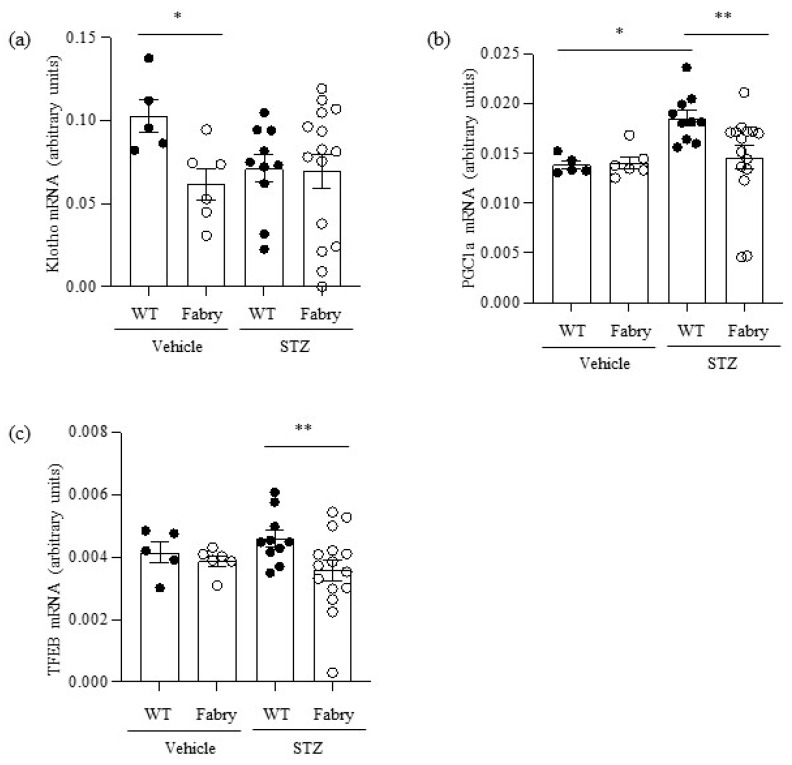
Impact of diabetes on the expression of kidney nephroprotective genes in Fabry mice. (**a**) Kidney *Klotho* mRNA, (**b**) *Pgc1α* mRNA, (**c**) *Tfeb* mRNA. * *p* < 0.05 vs. WT vehicle, ** *p* < 0.05 vs. WT diabetic. Data expressed as mean ± SEM of 5–15 animals per group. Significance (*p* < 0.05) was assessed by Student’s *t*-test for two groups of data and ANOVA for three or more groups with Bonferroni post-hoc correction.

Consistent with prior reports [21], non-diabetic Fabry and WT mice did not show significant differences in kidney function, histology, or gene expression, with the exception of a mild inflammation evidenced by F4/80 immunohistochemistry (Figure 2, Figure 3 and Figure 4). By contrast, DM promoted gene expression and/or histological evidence of kidney inflammation and fibrosis, which were observed in both WT and Fabry mice, as discussed below.

### 2.5. Interaction of DM and Fabry Disease on Kidney Inflammation and Fibrosis in Mice

Next, we explored the impact of DM on kidney inflammation and fibrosis in Fabry mice. MCP1 and RANTES are key chemokines upregulated during kidney injury. Kidney *Mcp1* and *Rantes* mRNA were significantly upregulated in diabetic WT mice compared to WT mice (Figure 2a,b), while values in diabetic Fabry mice did not significantly differ from those in diabetic WT mice (Figure 2a,b). The gene expression of the proinflammatory cytokine receptor *Fn14* was also significantly increased in diabetic WT mice compared to non-diabetic WT mice (Figure 2c) while diabetic Fabry mice did not differ from either non-diabetic Fabry mice or diabetic WT mice (Figure 2c). Of interest, kidney *Fn14* mRNA expression was very high in the two diabetic Fabry mice that developed kidney insufficiency and correlated with kidney function (Figure 2c,d). Kidney inflammation was assessed as infiltration by F4/80^+^ macrophages (Figure 2e). Both DM and Fabry disease promoted kidney infiltration by macrophages and the combination of DM and Fabry disease resulted in the highest inflammatory infiltrates: over 100 F4/80^+^ cells per hpf were only observed in mice with both conditions. Both kidney *Mcp1* and *Rantes* gene expression correlated with inflammatory infiltrates and between themselves (Figure S2).

Kidney fibrosis is a characteristic feature of both DKD and Fabry disease [22]. At the mRNA level, there was evidence of activation of fibrosis in diabetic WT mice and this was significantly milder in diabetic Fabry mice, as assessed by the kidney expression of the *Col1a2* and *Fn1* genes encoding interstitial fibrosis collagen and fibronectin, respectively (Figure 3a,b). Within the time frame studied, kidney fibrosis assessed by Sirius red staining, denoting collagen deposits, and interstitial fibronectin, representing extracellular matrix deposition, was only observed in some diabetic WT mice (Figure 3c,d). Similar to fibrosis gene expression changes, fibrosis was significantly milder in diabetic Fabry mice than in diabetic WT mice (Figure 3c,d). This contrasts with the level/expression of inflammatory markers (Fn14 and F4/80 macrophage infiltration) which were more severe in the diabetic Fabry mice. As was the case for chemokine expression, there was a good correlation between the kidney expression of *Col1a2* and *Fn1* and between these two genes and Sirius red quantification of fibrosis (Figure S3). 

### 2.6. Interaction of DM and Fabry Disease on the Expression of Kidney Protective Genes in Mice

So far, a lack of association has been observed between the severity of kidney inflammation and the severity of kidney fibrosis in diabetic Fabry and diabetic WT mice. The more severe kidney infiltration by leukocytes in diabetic Fabry mice than in diabetic WT mice could not be explained by differences in chemokine gene expression. Recently, attention has been focused on the role of the downregulation of kidney protective genes in the pathogenesis of kidney inflammation and disease [22,23]. Stress-inducing mediators such as high glucose levels or inflammatory cytokines may amplify kidney injury through further recruitment of inflammatory cells and downregulation of kidney protective factors such as Klotho or PGC1α [23,24,25]. Klotho has anti-inflammatory antifibrotic and antiaging properties and Klotho administration was protective in several experimental nephropathies [26,27,28,29,30,31,32,33,34]. PGC1α is a transcription factor master regulator of mitochondrial biogenesis that is downregulated during acute kidney injury [35,36,37,38,39,40]. PGC1α downregulation promotes kidney inflammation and sensitizes to kidney injury [41]. Finally, transcription factor EB (TFEB) is a master regulator of lysosomal biogenesis, autophagy, lysosomal exocytosis, lipid catabolism, energy metabolism and immune response [39,42,43,44,45].

Kidney Klotho mRNA was decreased in non-diabetic Fabry mice (Figure 4a). However, there was heterogeneity regarding kidney Klotho expression in diabetic Fabry mice, which included the two mice with strikingly low kidney Klotho expression. Western blot confirmed mRNA findings (Figure S4).

PGC1α mRNA was upregulated in diabetic WT mice compared to controls in what may be interpreted as an adaptive response to the additional energy stress imposed on proximal tubular cells to reabsorb large amounts of filtered glucose (Figure 4b). Interestingly, diabetic Fabry mice failed to upregulate PGC1α: kidney PGC1α mRNA was lower in diabetic Fabry mice than in diabetic WT mice and, additionally, it was strikingly decreased in two diabetic Fabry mice (Figure 4b). TFEB mRNA followed a similar pattern to PGC1α: a trend towards higher expression in diabetic WT mice and significant downregulation in diabetic Fabry mice compared to diabetic WT mice (Figure 4c).

Overall, kidneys from diabetic Fabry mice appear defective in the compensatory upregulation of kidney protective genes such as PGC1α and TFEB that may be upregulated in diabetic WT kidneys.

### 2.7. Diabetic Fabry Mice with Kidney Insufficiency

Two (12.5%) diabetic Fabry mice developed kidney insufficiency. Unlike other mice that died suddenly during follow-up, likely as a result of severe serum glucose excursions, these two mice looked sick and were sacrificed prematurely as per animal ethics committee guidelines and found to have developed kidney insufficiency. Interestingly, these two mice were outliers for certain features in the combination of both diabetic groups. This may provide insight into pathogenic mechanisms (Figure S5). Specifically, diabetic Fabry mice that developed kidney insufficiency had very high kidney gene expression for the upstream Gb3 pathway enzyme *Ugcg* and for the TWEAK receptor Fn14 (Figure S5a,b), and very suppressed expression of the kidney protective factor PGC1α (Figure S5c). 

## 3. Discussion

The main findings are that DM and Fabry disease may interact resulting in the modulation of kidney Gb3 synthesis pathways that may increase Gb3 availability through increased expression of *UGCG*, the gene encoding the upstream and rate-limiting enzyme glucosylceramide synthase, and limit the recruitment of compensatory kidney protective genes, potentially causing more severe inflammation and accelerated kidney failure.

In humans, diabetes is associated with changes in the kidney expression of genes encoding enzymes in the Gb3 pathway, including upregulation of *UGCG*, a therapeutic target in Fabry disease. Thus, glucosyl ceramide synthase inhibitors dramatically decrease Gb3 and lyso-Gb3 in Fabry patients [20]. Indeed, the increasing kidney *UGCG* expression in diabetic persons with more severe kidney disease was apparently “compensated” by increasing *GLA* expression as kidney disease got more severe, a “compensation” that would be absent in Fabry patients with defective *GLA* genes in which enzymatic activity would be absent or very reduced. Indeed, induction of DM in Fabry mice also resulted in increased kidney *Ugcg* gene expression in those mice that developed kidney insufficiency.

Insufficient activation of tissue protective genes such as Klotho, PGC1α and TFEB may favor end-organ damage [46,47]. Induction of DM in Fabry mice reproduced the kidney proinflammatory responses observed in diabetic WT mice. Additionally, Fabry mice failed to upregulate the nephroprotective genes PGC1α and TFEB in response to DM, potentially rendering the kidneys more sensitive to Fabry or DM-induced injury. While increased kidney inflammatory cell infiltration was observed in diabetic Fabry mice, this did not appear to be the result of increased kidney gene expression of the chemokines that were studied. However, PGC1α has anti-inflammatory actions in the kidney and was more severely downregulated when DM and Fabry disease coexisted, potentially accounting for the increased inflammation [41]. In this regard, the adverse impact of combining Fabry disease and DM appeared to be larger on inflammation than on fibrosis, potentially identifying a pathogenic molecular pathway to be explored in more detail: inflammation driven by loss of kidney protective factors.

While there were no significant differences in serum urea, creatinine, or proteinuria between diabetic Fabry and diabetic WT mice, 12% of diabetic Fabry mice developed kidney insufficiency which, in addition to increased plasma urea and creatinine, was characterized by a shared gene expression pattern of increased *Ugcg* and *Fn14* and suboptimal recruitment of the kidney protective factor *Pgc1α*. Given the design of the study, it remains unclear whether this implies that the combined gene expression pattern drives accelerated kidney disease in a subset of mice and what the trigger might be. Several potential links between these genes and tissue injury may be hypothesized (Figure S6) [23,41]. Interestingly, Fn14 and PGC1α have been previously linked in the pathogenesis of kidney injury, as TWEAK activation of Fn14 downregulates PGC1α leading to spontaneous inflammation and an increased severity of kidney injury [23,41]. Although there is less information on the interaction between UGCG and Fn14, both have been linked in biomarker panels in diverse clinical conditions [48,49]. Further studies are required to characterize how common is this phenotype in a longer follow-up or when older mice are studied. Indeed, older mice are prone to severe kidney injury, and this has been related to decreased expression of kidney protective factors, such as PGC1α and to exacerbated proinflammatory responses [50], i.e., the pattern observed in diabetic Fabry mice. Only a minority of male Fabry individuals develop severe kidney disease with late-onset genetic variants, such as N215S, in whom kidney failure is 100-fold more common than in the general population but only occurs in 3% of men with this variant [5]. Thus, the interaction with DM observed in mice with Fabry disease, which usually do not develop spontaneous kidney disease but may develop kidney insufficiency when DM is present, may be relevant to understanding the natural history of kidney disease in late-onset Fabry disease. 

The kidney phenotype of *Gla* deficient mice is histologically normal up to 48 weeks, despite lysosomal glycolipid deposits [21,50,51]. This is consistent with the long natural history of Fabry nephropathy in humans, of around 40 years from birth to kidney failure [52]. Although many biological processes are accelerated in short-lived animals, this is not the case for Fabry disease. However, mice are sensitive to Fabry nephropathy, as demonstrated when they overexpress Gb3 synthase on top of *Gla* deficiency, i.e., when glycolipid accumulation is larger [53].

Several limitations should be acknowledged. The short follow-up may not have fully grasped the full impact of the combination of Fabry disease and DM on the development of kidney insufficiency. However, ethics considerations precluded a longer experiment, given the mortality from kidney insufficiency of diabetic Fabry mice. Moreover, type 1 DM in young mice may not fully reflect type 2 DM in older individuals, i.e., the most common clinical scenario, which may result in a more severe phenotype. Backcrossing Fabry mice with db/db mice or other type 2 DM models and a long (>48 weeks) follow-up is beyond the scope of the current project but may provide further information in the future. Finally, gene expression may differ from protein expression. Despite the limitations, our work offers valuable multilevel (human systems biology, in vivo mouse studies) information on the interaction between DM and Fabry disease, uncovering evidence supporting that the combination may increase kidney disease severity in certain scenarios and having identified molecules that merit specific studies.

In conclusion, the combined presence of DM and Fabry disease may increase the severity of kidney disease through upregulation of the gene encoding the rate-limiting enzyme in Gb3 synthesis (*UGCG* encoding glucosyl ceramide synthase), downregulation of kidney protective factors and more severe inflammation. The emerging field of boosting kidney protection may be applicable to Fabry nephropathy developing in the presence of additional metabolic kidney stressors such as DM. In this regard, SGLT2 inhibitors decrease the urinary excretion of inflammatory cytokines and increase kidney protective molecules such as Klotho in patients with DM [25,54].

## 4. Materials and Methods

### 4.1. Animal Model

Procedures were conducted in accordance with the NIH Guide for the Care and Use of Laboratory Animals and were approved by the animal ethics committee of IIS-FJD (PROEX 036/16). We studied whether DM modifies the kidney phenotype of *Gla* knockout mice (Fabry mice, Jackson Lab, Bar Harbor, ME, USA) that contain a neo cassette replacing exon 3 and intron 3 of the *Gla* gene, abolishing gene expression. Insulin-deficient diabetes was induced in male Fabry or WT mice by administering intraperitoneally 125 mg/kg per day streptozotocin (STZ, Merck, Darmstadt, Alemania) for two consecutive days or vehicle [55]. Male mice were studied because Fabry is an X-linked disease. This means that because of random X chromosome inactivation, females are a mosaic of Fabry and healthy cells and the Fabry phenotype is not reproducible. Streptozotocin was dissolved in citrate buffer at pH 4.5. All streptozotocin-injected mice developed glycemia above 200 mg/dL. Severely hyperglycemic (blood glucose > 520 mg/dL) mice received NPH insulin (1.0–1.5 IU, Lilly USA, Indianapolis, IN, USA) daily to prevent weight loss and death [55]. After 30 days of DM, 16 h–fasted mice were anesthetized (100 mg/kg ketamine, (Ketalar, Pfizer, New York, NY, USA) and 15 mg/kg xylazine, (Xilagesic, Laboratorios Calier, Barcelona, Spain)). The intracardiac puncture was performed to collect blood in heparinized tubes which were subsequently centrifuged at 2500 rpm for 10 min to obtain plasma for biochemistry (urea, creatinine). Kidneys were perfused in situ with cold saline before removal. One kidney was snap-frozen in liquid nitrogen for RNA and protein studies and the other was fixed and paraffin-embedded for histological studies. Proteinuria was assessed by dipstick (Albustix, Siemens Healthcare, Erlangen, Germany). Two diabetic Fabry mice were sacrificed 17 and 27 days after administering the last streptozotocin dose because they looked sick and were found to have kidney insufficiency as defined by very high plasma creatinine and urea levels.

### 4.2. RT-qPCR

One microgram of RNA was isolated using Trizol (Invitrogen, Carlsbad, CA, USA) and reverse-transcribed with High Capacity cDNA Archive Kit.( Applied Biosystems, Foster City, CA, USA) Real-time quantitative PCR (RT-qPCR) was performed on an ABI Prism 7500 PCR system with pre-developed primer and probe assays (Applied Biosystems, Foster City, CA, USA) using the DeltaDelta Ct method, as previously described [56]. Expression levels are given as ratios to GAPDH expression.

### 4.3. Immunohistochemistry

Paraffin-embedded 3 µm thick tissue sections were processed for conventional histology or immunohistochemistry using the Envision detection kit (Dako, Glostrup, Denmark) according to the manufacturer’s instructions. For immunohistochemistry, sections were counterstained with Carazzi’s hematoxylin (PanReacAppliChem GmbH, Darmstadt, Germany). Primary antibodies were rat polyclonal anti-F4/80 antigen (1:50; Serotec, Oxford, UK) and mouse anti-fibronectin monoclonal (1:200, Chemicon, Temecula, CA, USA) antibodies. Negative controls included incubation with a non-specific immunoglobulin of the same isotype as the primary antibody. The total number of F4/80-positive macrophages was quantitated in 15 randomly chosen fields (200×) per kidney using Image-Pro Plus software (Media Cybernetics, Bethesda, MD, USA) and reported as number of positive cells/hpf. Staining was quantified in cortical tissue, as described [56].

For Sirius red staining, tissue sections were deparaffinized with xylene and graded concentrations of ethanol up to 70%, where slides stayed for 5 days at 4 °C. Direct Red 80 (Sigma-Aldrich, Merck, Darmstadt, Alemania, 365548) was dissolved in picrosirius acid (Sigma-Aldrich, Merck, Darmstadt, Germany, P6744) and incubated with tissue sections for 30 min at room temperature. Samples were dehydrated with a 100% ethanol wash and xylene. Slides were mounted in DPX medium (Merck, Darmstadt, Germany, 100579). The image was quantified with ImageProPlus software (Media Cybernetics, Bethesda, MD, USA), which allows selecting and calculating the area of pixels with similar colors. Results are shown as a percentage of positively stained area versus total quantified area from 10 fields per kidney (×200 magnification) [56]. Samples were examined in a blinded manner.

### 4.4. Data Mining

Information on the expression of genes encoding enzymes in the Gb3 pathway in human CKD transcriptomics datasets was obtained from Nephroseq v5 (http://v5.nephroseq.org/) accessed on 19 September 2022. A high-sensitivity approach was used, in which statistically significant differences (*p* < 0.05) in gene expression or correlation with analytical values were selected when representing a fold change >1.25 or an r value > 0.25 in DKD samples and their controls. Additionally, the Kidney Interactive Transcriptomics (KIT) webpage (http://humphreyslab.com/SingleCell/displaycharts.php; accessed on 19 May 2023) was used to identify individual cell types that may show differential expression of genes encoding Gb3 pathway enzymes in human DKD [57,58].

### 4.5. Statistics

Statistical analysis was performed using GraphPad Prism Software 8 (GraphPad Software, San Diego, CA, USA). Results are expressed as mean ± SEM. Significance (*p* < 0.05) was assessed by Student’s t-test for two groups of data and ANOVA for three or more groups with Bonferroni post-hoc correction. Pearson correlation was used to assess relationships between two continuous variables.

## Data Availability

The data used and/or analysed during the current study are available from the corresponding author on reasonable request.

## Supplementary Materials

The following supporting information can be downloaded at: https://www.mdpi.com/article/10.3390/ijms242115853/s1. Reference [59] is cited in the supplementary materials.
