# Supplementary material for: Interaction of Fabry Disease and Diabetes Mellitus: Suboptimal Recruitment of Kidney Protective Factors
Source: Int J Mol Sci. 2023 Nov 1;24(21):15853. doi: 10.3390/ijms242115853 (PMC10650640; doi:10.3390/ijms242115853)
Supplement: Supplementary file 1 [file ijms-24-15853-s001.zip › ijms-2661069-supplementary.pdf]

Suppl Figure S1

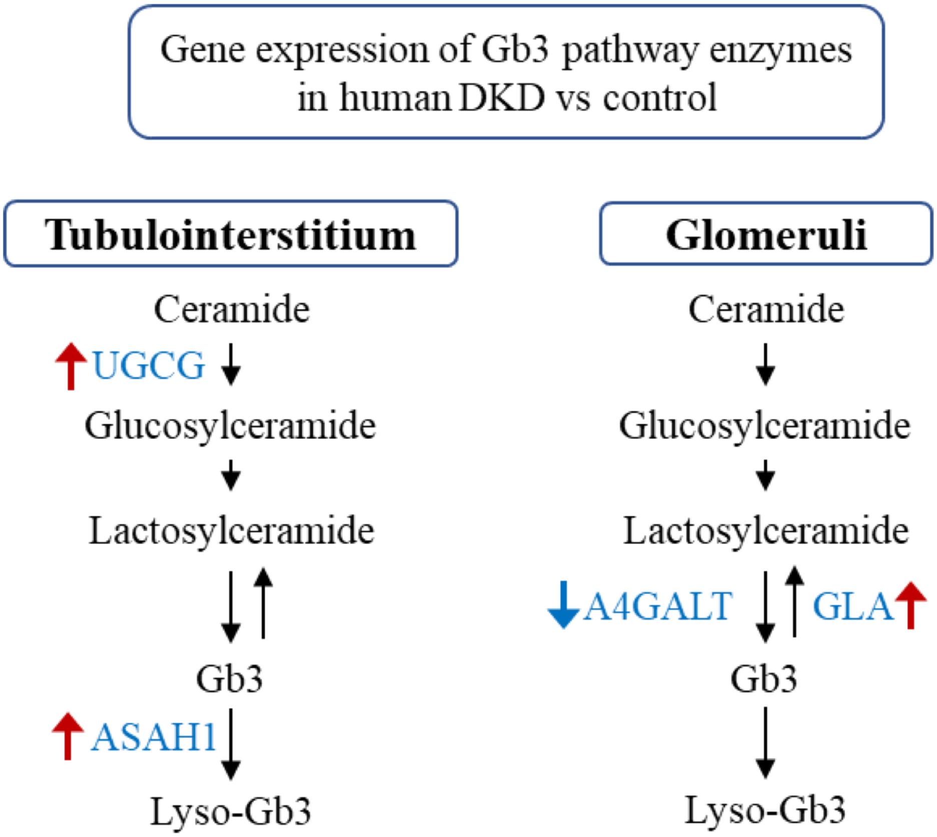

Suppl Figure S2

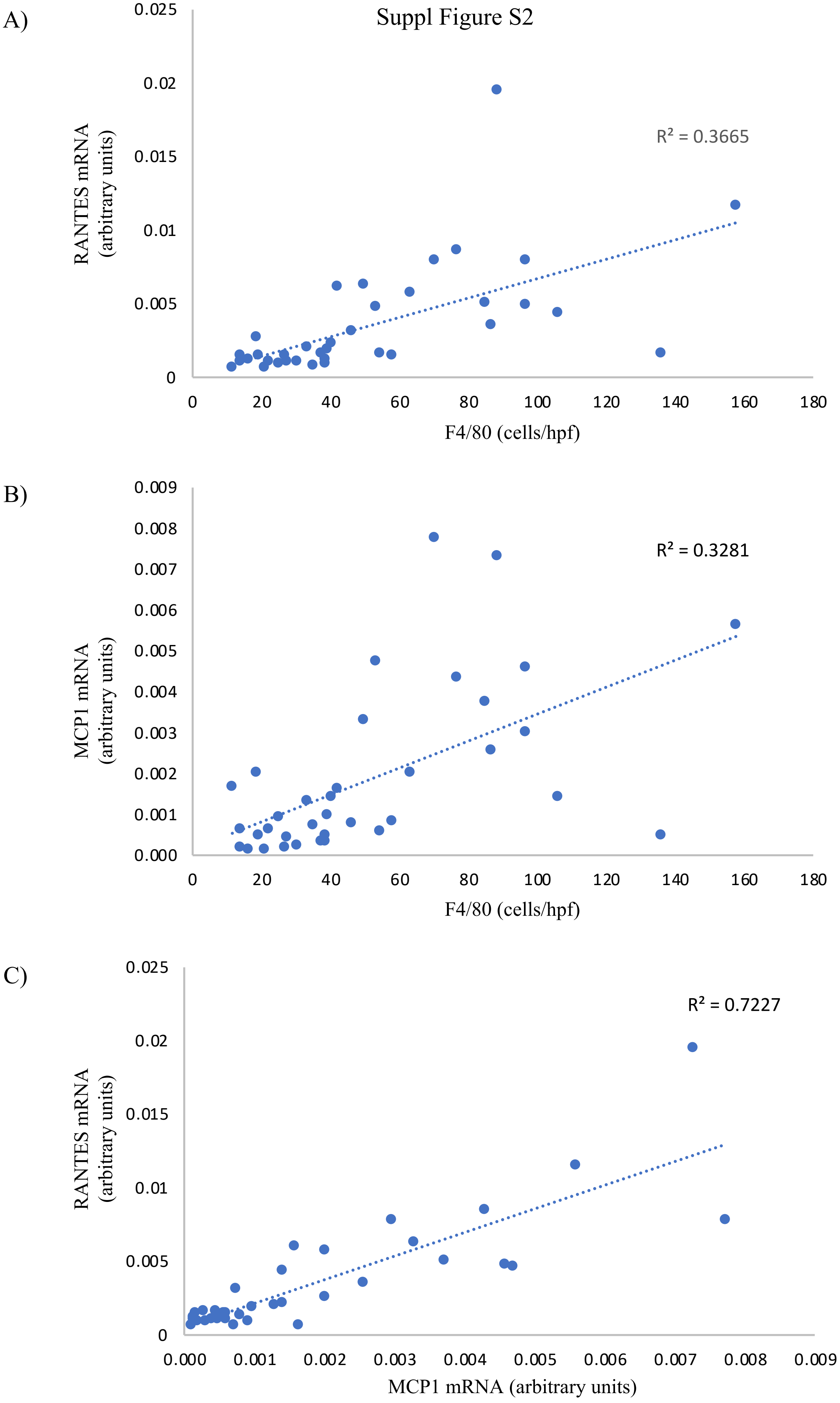

Suppl Figure S3

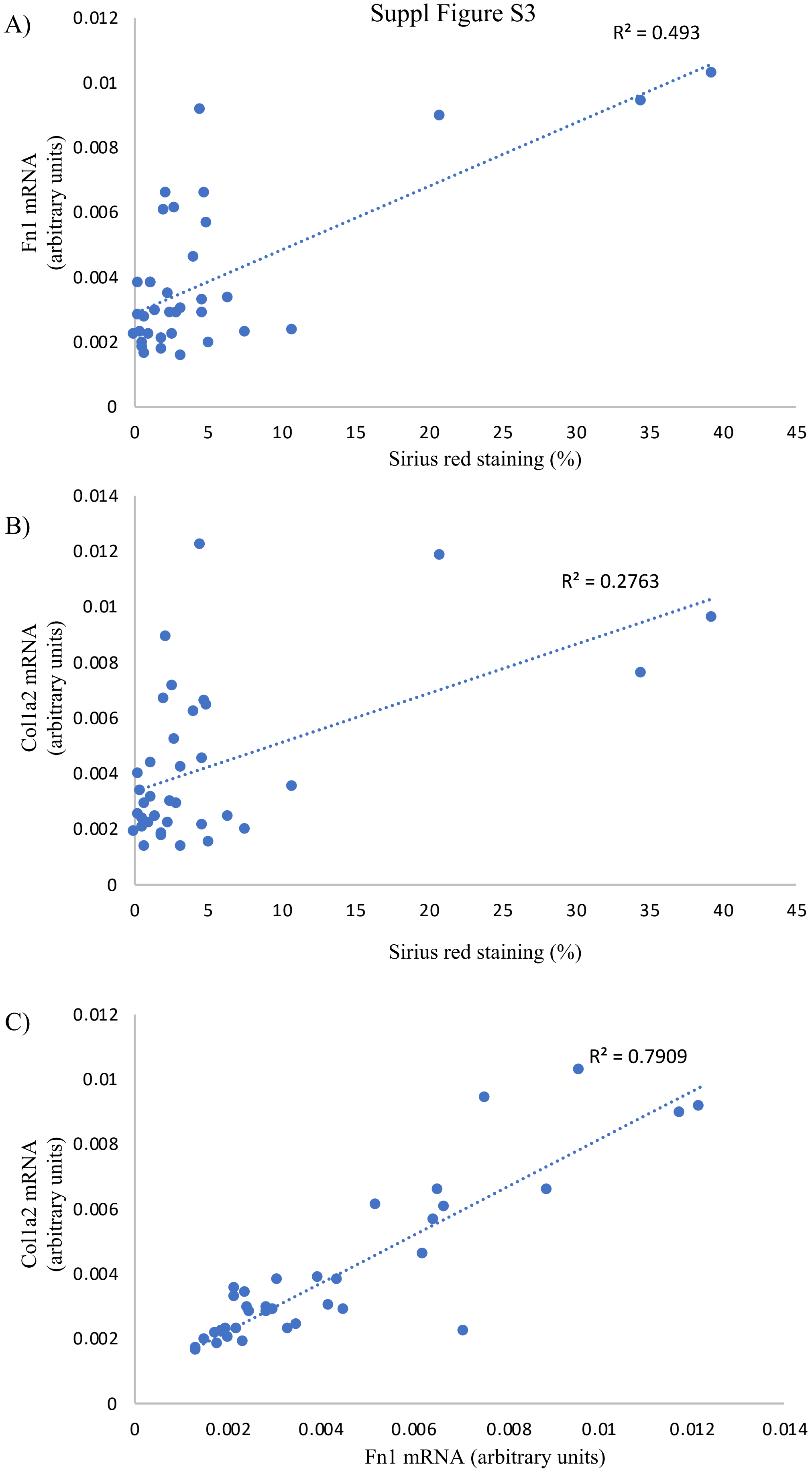

Suppl Figure S4

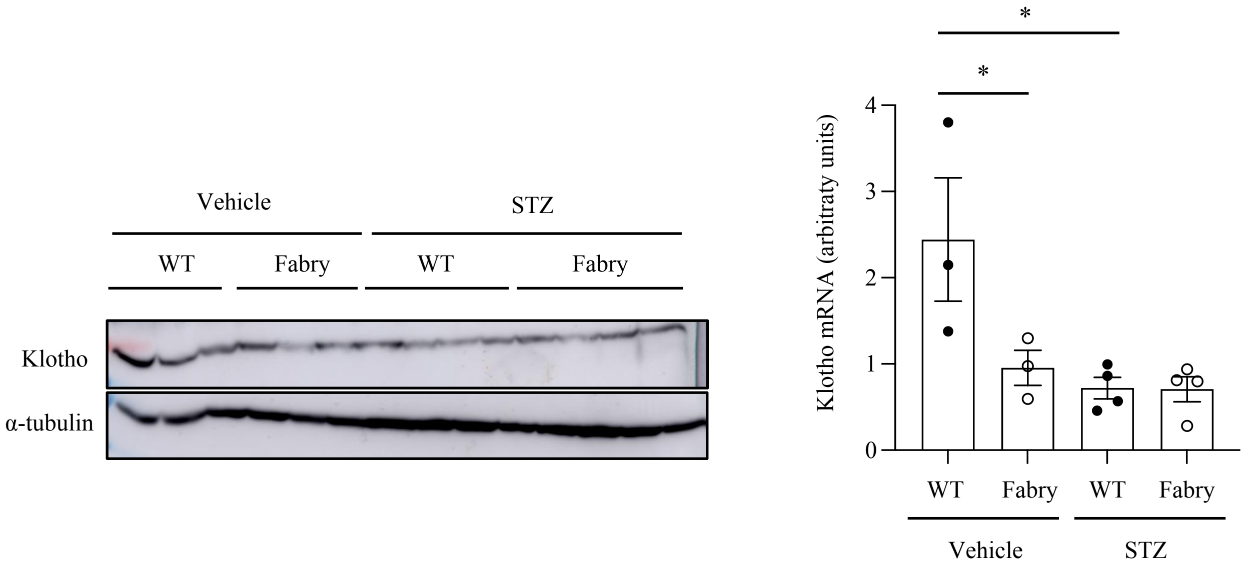

Suppl Figure S5

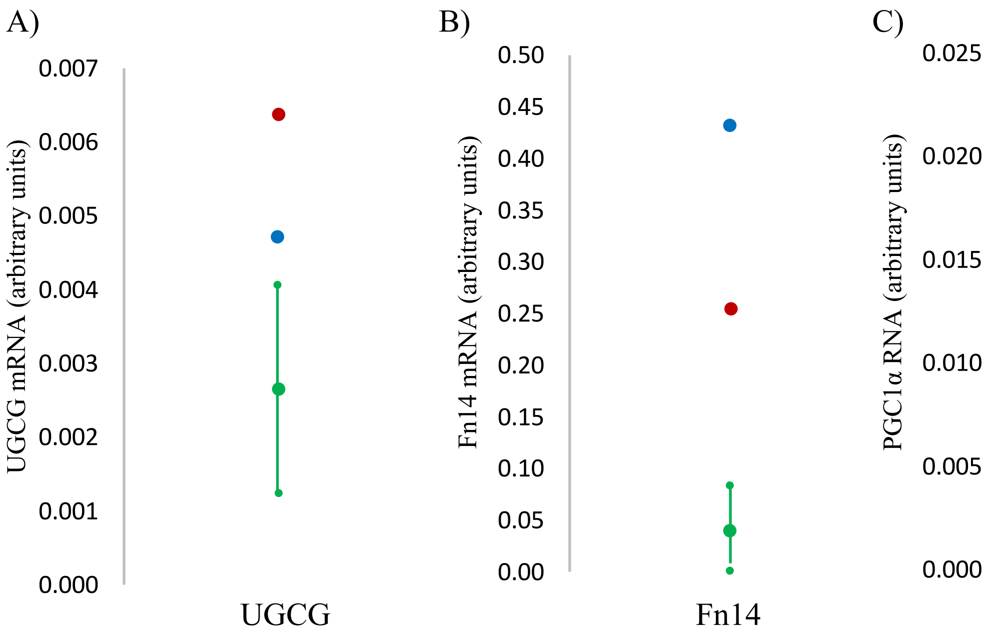

Suppl figure S6

A) Lineal relationship between key molecules

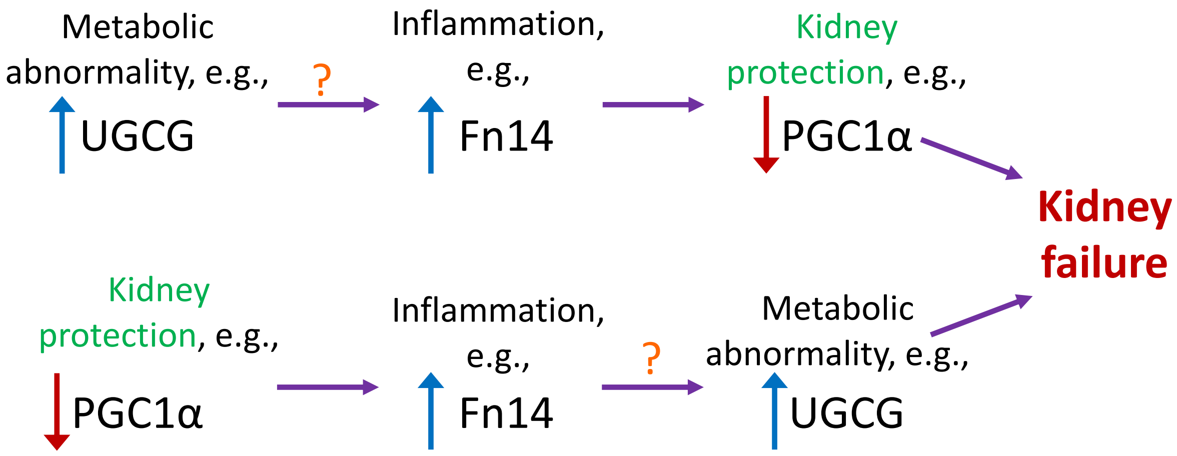

B) Vicious circle relationship between key molecules

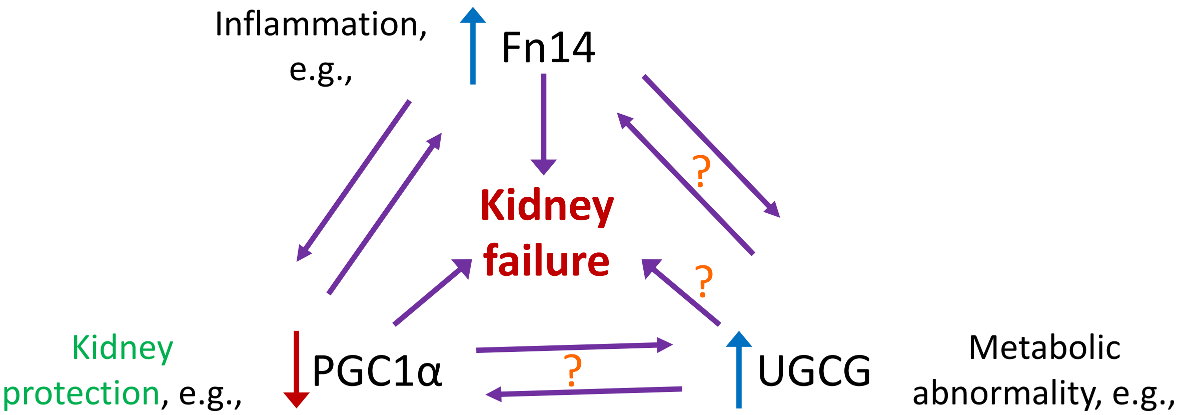

SUPPLEMENTARY MATERIAL

Supplementary methods

Animal model

Procedures were conducted in accordance with the NIH Guide for the Care and Use of Laboratory Animals and were approved by the animal ethics committee of IIS-FJD (PROEX 036/16). We studied whether DM modifies the kidney phenotype of *Gla* knockout mice (Fabry mice, Jackson Lab) that contain a neo cassette replacing exon 3 and intron 3 of the *Gla* gene, abolishing gene expression. Insulin-deficient diabetes was induced in male Fabry mice or wild type (WT) mice by intraperitoneal streptozotocin (125 mg/kg per day for 2 days) or vehicle. Streptozotocin was dissolved in citrate buffer at pH 4.5. All mice developed glycemia above 200 mg/dl. Severely hyperglycemic (blood glucose >520 mg/dl) mice received NPH insulin (1.0–1.5 IU) daily to prevent weight loss and death[1,2]. After 30 days of DM, plasma samples were collected for biochemistry (urea, creatinine), and kidneys were perfused in situ with cold saline before removal. One kidney was snap-frozen in liquid nitrogen for RNA and protein studies and the other fixed and paraffin-embedded for histological studies. Proteinuria was assessed by dipstick (Albustix, Siemens). Two diabetic Fabry mice were sacrificed early (days 17 and 27 after administering the last streptozotocin injection) because they looked sick and were found to have kidney failure.

RT-qPCR

One µg RNA was isolated using Trizol (Invitrogen UK) and reverse-transcribed with High Capacity cDNA Archive Kit. Real-time PCR was performed on an ABI Prism 7500 PCR system with pre-developed primer and probe assays (Applied Biosystems, Foster City, CA) using the DeltaDelta Ct method, as previously described [3]. Expression levels are given as ratios to GAPDH expression.

Western blot

Tissue samples were homogenized in lysis buffer, then separated by 10% SDS-PAGE under reducing conditions and transferred to PVDF membranes (Millipore, Bedford, MA, USA), blocked with 5% skimmed milk in PBS/0.5% v/v Tween 20 for 1 h, and washed with PBS/Tween 25. Primary antibody was rabbit polyclonal anti-Klotho (1:1000, Calbiochem, La Jolla, California) while anti-α-tubulin (1:5000, Sigma, St. Louis, MO, USA) was used to account for minor differences in loading.

Immunohistochemistry

Paraffin-embedded 3 µm thick tissue sections were processed for conventional histology or immunohistochemistry using the Envision detection kit (Dako) according to the manufacturer's instructions. For immunohistochemistry, sections were counterstained with Carazzi`s hematoxylin. Primary antibodies were rat polyclonal anti-F4/80 antigen (1:50; Serotec, Oxford, UK). Negative controls included incubation with a non-specific immunoglobulin of the same isotype as the primary antibody. The total number of F4/80-positive macrophages was quantitated in 15 randomly chosen fields (200x) per kidney using Image-Pro Plus software (Media Cybernetics, Bethesda, MD) and reported as number of positive cells/hpf. Staining was quantified in cortical tissue, as described [3].

For Sirius red staining, tissue sections were deparaffinized with xylene and graded concentrations of ethanol up to 70%, where slides stayed for 5 days at 4°C. Direct Red 80 (Sigma-Aldrich, 365548) was dissolved in picrosirius acid (Sigma-Aldrich, P6744) and incubated with tissue section for 30 min at room

temperature. Samples were dehydrated with a 100% ethanol wash and xylene. Slides were mounted in DPX medium (Merck, 100579). Image was quantified with ImageProPlus software (Media Cybernetics, Bethesda, MD), that allows selecting and calculating the area of pixels with similar color. Results are shown as percentage of positive stained area versus total quantified area from 10 fields per kidney (x200 magnification) [3]. Samples were examined in a blinded manner.

Supplementary figures

Figure S1. Impact of diabetes on genes encoding enzymes in the Gb3 pathway in wild type and Fabry mice. Scheme of Gb3 synthesis and metabolism: glucosylceramide synthase encoded by UGCG, Gb3 synthase encoded by A4GALT, acid ceramidase encoded by ASAH1, and alpha-galactosidase A encoded by GLA. Arrows present a summary of changes in gene expression, as presented in detail in table S1.

Figure S2. Relationship between markers of kidney inflammation in mice with diabetes and/or Fabry disease. A) Correlation between kidney RANTES mRNA expression and F4/80$^+$ inflammatory cell infiltration. **B)** Correlation between kidney MCP1 mRNA expression and F4/80$^+$ cell infiltration. **C)** Correlation between kidney RANTES and MCP1 mRNA expression. All p values <0.05.

Figure S3. Relationship between markers of kidney fibrosis in mice with diabetes and/or Fabry disease. A) Correlation between kidney *Fn1* mRNA expression and Sirius red staining for fibrosis. **B)** Correlation between kidney *Col1a2* mRNA expression and Sirius red staining for fibrosis. **C)** Correlation between kidney Col1a2 and *Fn1* mRNA expression. All p values <0.05.

Figure S4. Kidney Klotho protein. Western blot of kidney Klotho. *p<0.05 vs WT vehicle. Data expressed as mean±SEM of 3-4 animals per group.

Figure S5. Kidney gene expression for outliers in diabetic Fabry mice that developed kidney failure. Three genes were expressed in the kidneys of diabetic Fabry mice with kidney failure to levels outside the 95% confidence interval (green) of the other 13 mice in the group. **A)** UGCG mRNA. **B)** Fn14 mRNA **C)** PGC1α mRNA. Mice with kidney failure are color-coded in blue and red.

Figure S6. Hypothetical scenarios that may underlie the interaction between DM and Fabry disease leading to kidney failure. A) Linear scenario. The specific molecules implicated, and the cause-and-effect relationships may differ from the examples shown. It is well established that inflammation decreases the expression of kidney protective genes and that the loss of some of these genes (e.g., Klotho, PGC1α) promotes kidney inflammation. However, the potential role for UGCG in a *Gla*-deficient context remains unstudied. **B)** Vicious circle scenario. This scenario may better explain the rapidly progressive course of kidney failure in some mice. As for the linear scenario, the relationship between events in diabetes and in Fabry disease remains at this point hypothetical and should be validated in interventional studies. In both cases, the potential triggers have not been identified. While kidney failure was uncommon in our experimental model (12%), this is also the case for kidney failure in persons with *GLA* gene variants, such as N215S, causing late onset Fabry disease: they are not usually associated with kidney failure: just 15% of them developed kidney failure in a large study but this rate is 100-fold higher than in the general population [4].

Supplementary Tables

Table S1. Kidney mRNA expression of genes encoding enzymes in the Gb3 pathway in DKD.

A) Fold-change kidney (TI: Tubulointerstitium, Glom: Glomeruli) mRNA expression is shown in human DKD compared to control kidneys, according to the Nephroseq database of kidney transcriptomics studies. Search criteria were fold change >1.25, p value <0.05.

Gene	Tissue	Fold-change	p value	Ref
UGCG	Human Diabetes TI	1.798	0.002	http://v5.nephroseq.org/
	Human Diabetes TI	2.136	0.01	[5]
	Human Diabetes TI	1.396	0.018	[6]
A4GALT	Human Diabetes Glom	-1.295	5.23E-05	[6]
ASAH1	Human Diabetes TI	1.256	0.007	[8]
GLA	Human Diabetes Glom	1.407	4.11E-08	[6]

B) Correlation of diabetic kidney (TI: Tubulointerstitium, Glom: Glomeruli) mRNA expression with eGFR (assessed by the creatinine-based MDRD equation unless otherwise specified) in human DKD is shown, according to the Nephroseq database of kidney transcriptomics studies. Search criteria were r value >0.25, p value <0.05. *A4GALT* did not show any significant association with DKD or correlation with eGFR.

Gene	Sample	r value	p value	Ref
UGCG	TI	-0.676	0.022	[8]
	TI (Cockroft-Gault)	-0.822	0.002	[8]
ASAH1	Glom	0.716	0.030	[5]
	TI	0.444	0.038	[5]
GLA	Glom	-0.679	0.044	[5]
	TI	-0.716	0.046	http://v5.nephroseq.org/

Table S2. Gb3 metabolic pathway gene expression in human DKD (n=3) as compared to control kidneys (n=3). Data were obtained from table S5 in reference [9].

**UGCG**	**DM**	**Control**	**avg_logFC**	**Ratio DM/control**	**p value**	**p value adjusted**
PCT	0.447	0.249	0.379	1.795	8.3E-55	3E-50
DCT	0.277	0.243	0.159	1.140	3.0E-02	1E+00
Endo	0.298	0.211	0.316	1.412	2.2E-04	1E+00
Mes	0.217	0.143	0.326	1.517	1.9E-02	1E+00
Leuko	0.199	0.024	0.796	8.292	5.9E-03	1E+00
**ASAH1**						
PCT	0.292	0.17	0.049	1.718	3.5E-25	1E-20
PEC	0.165	0.108	0.096	1.528	9.2E-03	1E+00
CD-PC	0.215	0.249	-0.071	0.863	1.3E-02	1E+00
**A4GALT**						
PCT	0.185	0.143	-0.055	1.294	1.2E-04	1E+00
PEC	0.125	0.187	-0.190	0.668	8.6E-03	1E+00
CD-ICA	0.091	0.132	-0.104	0.689	6.3E-03	1E+00

Supplementary References

1 Sanchez-Niño MD, Sanz AB, Ihalmo P *et al.* The MIF receptor CD74 in diabetic podocyte injury. *J Am Soc Nephrol* 2009; **20**: 353–362.

2 Sanchez-Niño MD, Sanz AB, Lorz C *et al.* BASP1 promotes apoptosis in diabetic nephropathy. *J Am Soc Nephrol* 2010; **21**: 610–621.

3 Valiño-Rivas L, Cuarental L, Ceballos MI *et al.* Growth differentiation factor-15 preserves Klotho expression in acute kidney injury and kidney fibrosis. *Kidney Int* 2022; **101**: 1200–1215.

4 Germain DP, Brand E, Burlina A *et al.* Phenotypic characteristics of the p.Asn215Ser (p.N215S) GLA mutation in male and female patients with Fabry disease: A multicenter Fabry Registry study. *Mol Genet genomic Med* 2018; **6**: 492–503.

5 Woroniecka KI, Park ASD, Mohtat D, Thomas DB, Pullman JM, Susztak K. Transcriptome analysis of human diabetic kidney disease. *Diabetes* 2011; **60**: 2354–69.

6 Ju W, Nair V, Smith S *et al.* Tissue transcriptome-driven identification of epidermal growth factor as a chronic kidney disease biomarker. *Sci Transl Med* 2015; **7**: 316ra193.

7 Hodgin JB, Nair V, Zhang H *et al.* Identification of cross-species shared transcriptional networks of diabetic nephropathy in human and mouse glomeruli. *Diabetes* 2013; **62**: 299–308.

8 Schmid H, Boucherot A, Yasuda Y *et al.* Modular activation of nuclear factor-kappaB transcriptional programs in human diabetic nephropathy. *Diabetes* 2006; **55**: 2993–3003.

9 Wilson PC, Wu H, Kirita Y *et al.* The single-cell transcriptomic landscape of early human diabetic nephropathy. *Proc Natl Acad Sci U S A* 2019; **116**: 19619–19625.
